# Case report: Peritumoral hepatic steatosis in a patient with a metastatic somatostatin-producing oligosymptomatic neuroendocrine neoplasm

**DOI:** 10.3389/fonc.2022.1013017

**Published:** 2022-12-02

**Authors:** Yuming Shao, Yang Gui, Yuejuan Cheng, Jia Xu, Xiaoyan Chang, Ke Lv

**Affiliations:** ^1^ Department of Ultrasound, Peking Union Medical College Hospital, Chinese Academy of Medical Sciences and Peking Union Medical College, Beijing, China; ^2^ Department of Medical Oncology, Peking Union Medical College Hospital, Chinese Academy of Medical Sciences and Peking Union Medical College, Beijing, China; ^3^ Department of Radiology, Peking Union Medical College Hospital, Chinese Academy of Medical Sciences and Peking Union Medical College, Beijing, China; ^4^ Department of Pathology, Peking Union Medical College Hospital, Chinese Academy of Medical Sciences and Peking Union Medical College, Beijing, China

**Keywords:** pancreas, neuroendocrine neoplasm, somatostatin, peritumoral steatosis, case report

## Abstract

Neuroendocrine neoplasms (NENs) comprise a heterogeneous collection of tumors derived from various neuroendocrine cells and are divided into functioning NEN and non-functioning NEN. Some NENs present with mild symptoms and can secrete somatostatin. These neoplasms are known as somatostatin-producing oligosymptomatic NENs. In this report, we describe a case of metastatic somatostatin-producing oligosymptomatic NEN with peritumoral hepatic steatosis and review the relevant literature. The patient was a 45-year-old woman who presented with mild steatorrhea and melena. A computed tomography scan revealed an enlarged pancreas protruding into the duodenum. Pathology after total pancreatectomy showed a grade 2 pancreatic NEN with positive somatostatin immunostaining. Enlarging masses on the liver were observed after the operation. Ultrasound examination revealed several lesions in the liver, with inner hypoechoic areas that showed rapid enhancement and fast washout on contrast-enhanced ultrasonography and with outer hyperechoic areas with continuous iso-enhancement. Therefore, the inner hypoechoic areas seen on contrast-enhanced ultrasonography were suspected to be true metastases. A biopsy confirmed this suspicion and indicated that the outer areas were peritumoral liver steatosis. This case highlights the importance of the imaging pattern described in this report for accurate diagnosis of metastatic NEN to avoid incorrect estimation of tumor size or a missed diagnosis on biopsy.

## Introduction

As found in various organs, neuroendocrine neoplasms (NENs) are a group of neural crest tumors, at least 70% of which are of gastroenteropancreatic origin (1). Somatostatin-producing oligosymptomatic NEN is a subtype of pancreatic NEN. In this report, we describe a patient who had somatostatin-producing oligosymptomatic NEN with metastasis to the liver and peritumoral hepatic steatosis. This is the first report on the unique pathological and imaging features of this specific subtype of tumor.

## Case presentation

**Figure 1:**
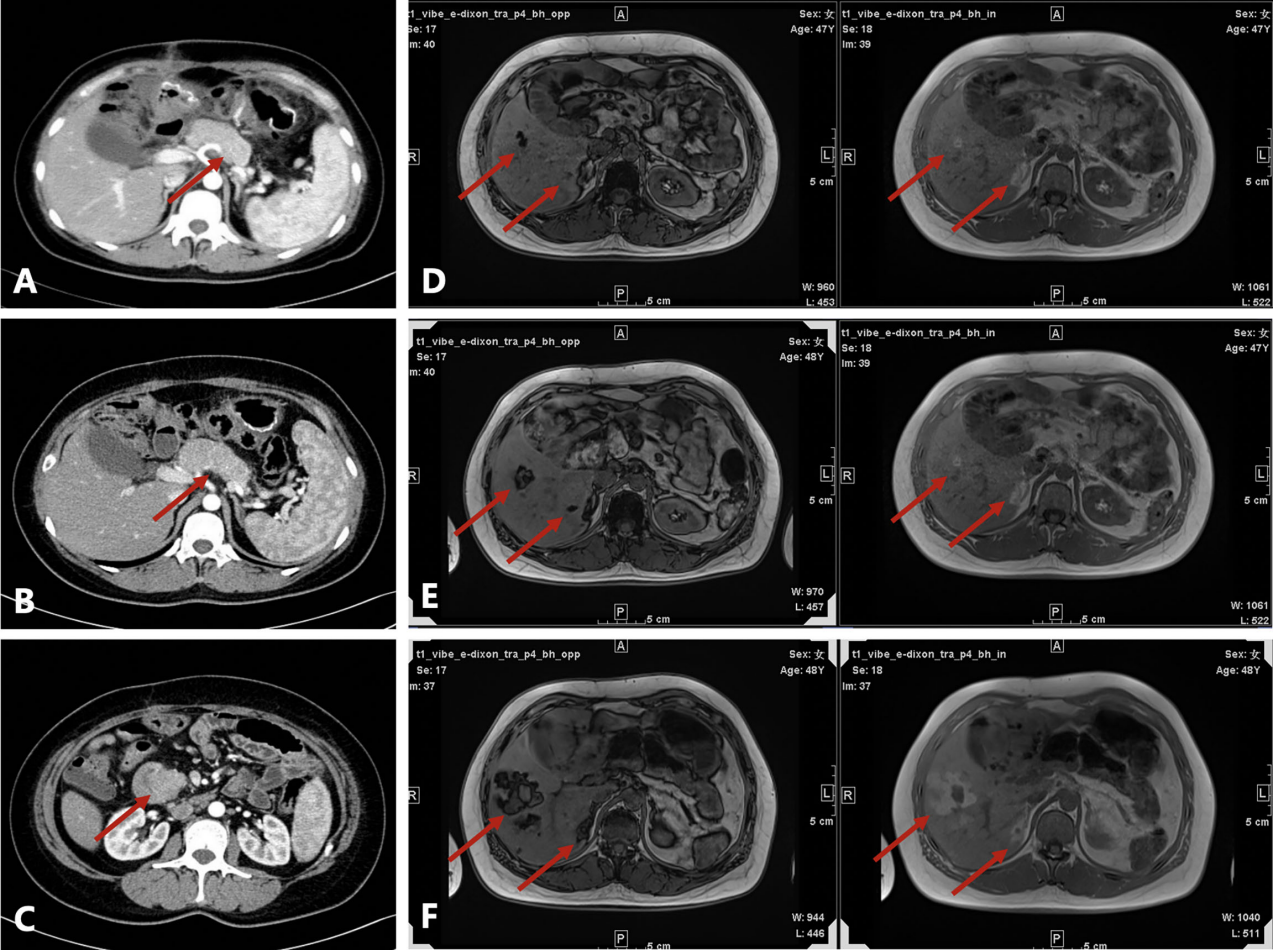
Radiological evaluation before and after surgery. **(A)** Irregularly shaped pancreas seen on contrast-enhanced computed tomography (CT) at the first admission. **(B)** Enlarged pancreas on contrast-enhanced CT before surgery. **(C)** Head of the pancreas protruding into the duodenum on contrast-enhanced CT before surgery. **(D–F)** Axial T1-weighted in-phase and out-of-phase images obtained during follow-up (D, 1.5 years after surgery; E, 2 years after surgery; F, 2.5 years after surgery). There were several patchy and nodular high-signal lesions in the right lobe of the liver on the in-phase image. Note a significant signal drop in these lesions on the opposed-phase image. The lesions gradually enlarged during follow-up. A nodule inside the largest lesion was detected in the last follow-up scan.

**Figure 2:**
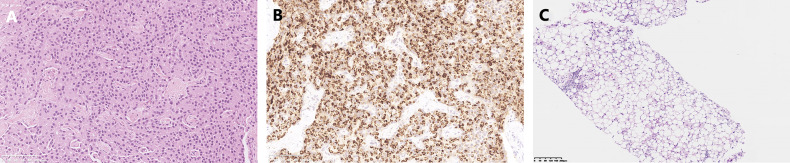
Pathological results. **(A)** Hematoxylin-eosin staining of the pancreatic neuroendocrine neoplasm. **(B)** Somatostatin immunostaining. **(C)** Biopsy of the liver metastases.

A 45-year-old Chinese woman with mild steatorrhea and melena was admitted to our hospital after pathological examination of a biopsy of the pancreas confirmed grade 2 pancreatic NEN. The patient had no other signs or manifestations, such as abdominal pain, weight loss, jaundice, palpitations, hunger, reflux, or skin rash. Computed tomography (CT) examination of the abdomen and pelvis revealed an irregularly shaped pancreas. No abnormality was apparent in the liver or other organs (**Figure 1A**). Total pancreatectomy had originally been recommended but was refused by the patient. Eight months later, the melena worsened. A CT examination showed a more enlarged pancreas, part of which was protruding into the duodenum (**Figures 1B, C**). Physical examination did not reveal any important clinical findings. Preoperative laboratory investigations, including for CA199, CEA, CA125, insulin, C-peptide, fasting serum glucose, somatostatin, glucagon, and gastrin, were normal. Screening of the parathyroid glands and adenohypophysis was negative. The family and psychosocial history was normal. Total pancreatectomy and splenectomy were performed *via* an open approach, and R0 resection was achieved. The pathological results were consistent with grade 2 pancreatic NEN with 2 mitoses/2 mm^2^ and a Ki-67 proliferation index of 5%. Immunostaining of chromogranin A, synaptophysin, and somatostatin was positive, but immunostaining of insulin, gastrin, and glucagon was negative (**Figures 2A, B**). In view of the clinical findings not being strong or distinctive enough for somatostatinoma and mild steatorrhea being the only sign present early in the clinical course, the final diagnosis was somatostatin-producing oligosymptomatic NEN.

**Figure 3:**
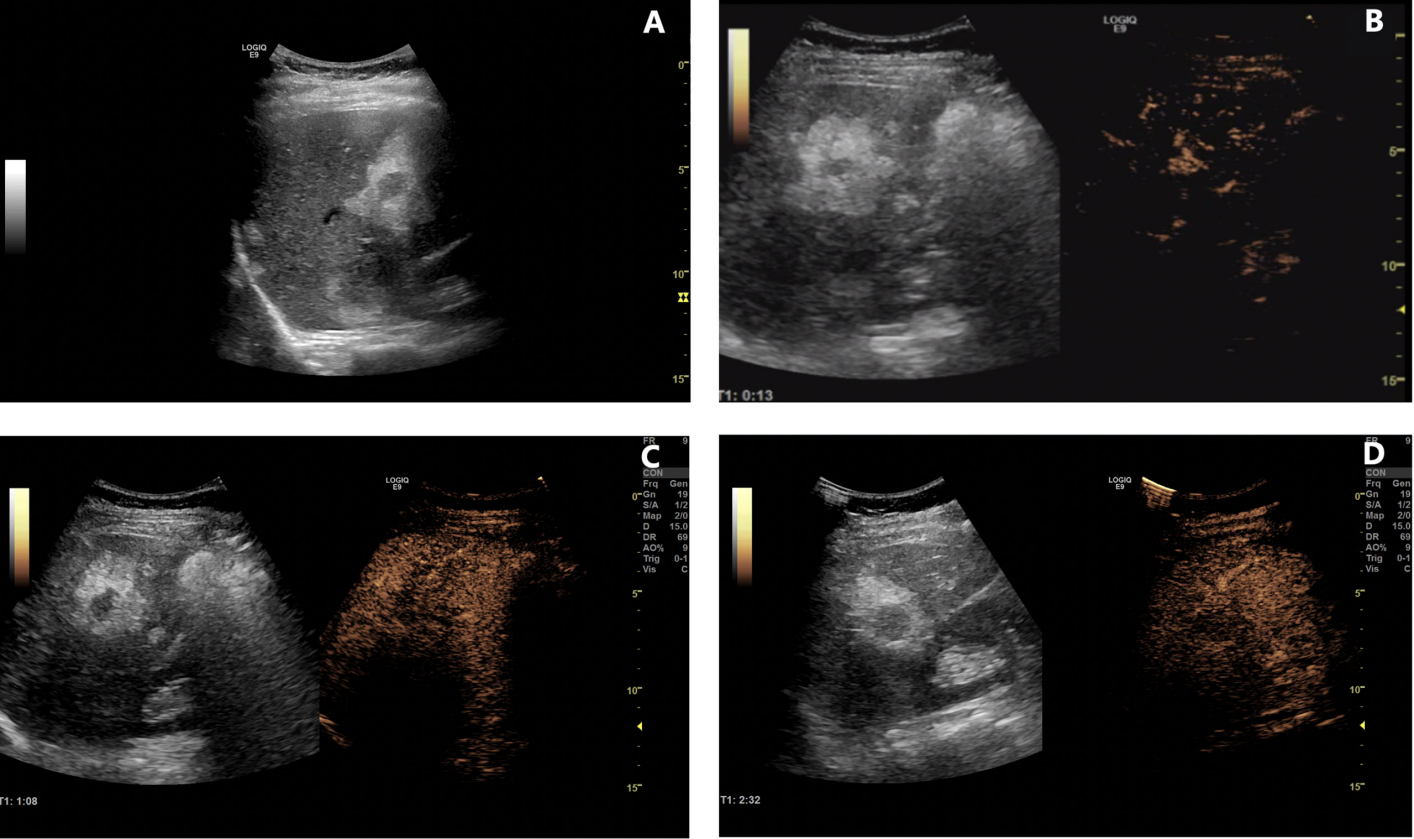
Contrast-enhanced ultrasonography of lesions in the liver. **(A)** B-mode ultrasound examination revealed several lesions with an inner hypoechoic area and outer hyperechoic area. **(B–D)** Contrast-enhanced ultrasonographic scans [**(B)**, 13 s in the arterial phase; **(C)**, 68 s in the portal venous phase; **(D)**, 152 s in the delayed phase] showed rapid enhancement in the arterial phase, early washout in the portal venous phase, and hypo-enhancement in the delayed phase in the inner hypoechoic area. However, the peripheral area was continuously iso-enhancing or hypo-enhancing.

The patient was followed up regularly after surgery at 3-month intervals with magnetic resonance imaging (MRI) of the abdomen every 6 months. At 1.5 years postoperatively, multiple small patchy and nodular lesions with opposite signals on axial T1-weighted in-phase and out-of-phase MRI were detected in the liver for the first time (**Figure 1D**). In view of the indeterminate nature of these lesions at that time, continued follow-up was recommended. The volumes of the lesions continued to increase after a further 6 months and 1 year (**Figures 1E, F**). Given that the lesions showed opposite signals on in-phase and out-of-phase T1-weighted MRI and were enlarging during follow-up, metastatic tumor with fatty changes was strongly suspected. We then performed a comprehensive ultrasonographic investigation of the liver lesions. A conventional ultrasound scan revealed several irregularly shaped hyperechoic lesions in the liver. The largest lesion measured 6.7 × 3.9 cm and was located in the right lobe. A hypoechoic area with a diameter of 2.2 cm was observed inside this hyperechoic lesion (**Figure 3A**). Contrast-enhanced ultrasonography (CEUS) was then performed to confirm the exact location of the lesion and to avoid the possibility of a false-negative biopsy result. CEUS of the inner hypoechoic area showed rapid enhancement in the arterial phase and early washout in the portal venous phase with hypo-enhancement in the delayed phase. However, continuous iso-enhancement or hypo-enhancement was seen in the peripheral area (**Figures 3B–D**). The patient had no discomfort, and her liver function tests were normal. A real-time ultrasound-guided biopsy was performed to ensure extraction of both the peripheral hyperechoic and inner hypoechoic areas and to clarify the nature of the lesions. Histologic evaluation showed a small number of NEN cells and peritumoral liver cells with fatty change (**Figure 2C**).

**Figure 4:**
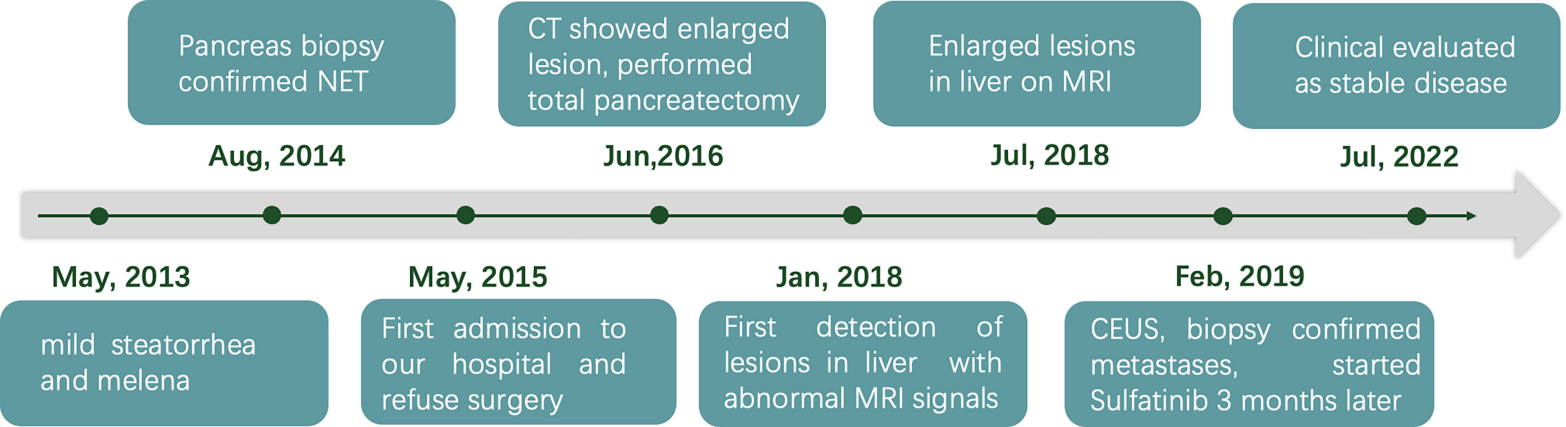
Timeline showing the course of the disease.

Therefore, a diagnosis of liver metastasis of NEN was made. Three months later, the patient was started on sulfatinib, a small-molecule drug that targets the vascular endothelial growth factor and fibroblast growth factor receptors, as part of a clinical trial. The patient was followed up intensively at monthly intervals. Grade 1 hypertension and abnormal liver function were documented as adverse effects. Clinical evaluation in July 2022 (40 months after biopsy of the liver lesions) revealed stable disease. Timeline showing the course of the disease is in **Figure 4**.

## Discussion

This report describes a patient with metastatic NEN to the liver who had unique pathological and imaging features. The hepatic metastases were surrounded by liver cells showing fatty changes. Ultrasonography showed that these lesions contained a hypoechoic area in the center of a hyperechoic area. CEUS was able to distinguish the actual boundary of the metastases and to guide liver biopsy. To the best of our knowledge, this is the first report of metastatic somatostatin-producing oligosymptomatic NEN with peritumoral liver steatosis.

Pancreatic NENs are divided into functioning and non-functioning tumors. Functioning NENs can secrete a large amount of hormones, causing specific clinical hormone hypersecretion syndromes, including insulinoma, glucagonoma, somatostatinoma, gastrinoma, and VIPoma as well as serotonin-producing tumors and adrenocorticotropic hormone-producing tumors. Non-functioning tumors are not associated with clinical hormone hypersecretion syndromes. However, some may still produce biogenic hormones and stain on immunochemistry. The levels of these hormones, which include glucagon, pancreatic polypeptide, somatostatin, and chromogranin, are insufficient to cause clinical symptoms (2). Tumors that demonstrate immunohistochemical labeling with somatostatin but are not associated with symptoms of somatostatinoma syndrome, such as diabetes, cholelithiasis, or diarrhea/steatorrhea, should be classified as somatostatin-producing well-differentiated NEN rather than somatostatinoma (3). The patient in this report presented with only mild steatorrhea, had no other obvious clinical manifestations of somatostatinoma syndrome, and did not have abnormal serum hormone levels. Therefore, this case should be designated as somatostatin-producing oligosymptomatic NEN.

Although the imaging features of liver metastases vary because of the heterogenous origins of the primary tumors, the ultrasonogram in this case showed features that are not often encountered, which made diagnosis difficult. The outer hyperechoic area may be mistaken for tumor tissue and the inner hypoechoic area may be interpreted as liquidation, necrosis, or mucin secretion. Under these circumstances, the tumor volume may be overestimated or the diagnosis missed if the biopsy extracts tissue only from the outer hyperechoic area. However, CEUS may have unique advantages that overcome the abovementioned problems to some extent. In this case, the enhancement pattern in the outer area seemed to indicate normal or relatively normal hepatic tissue, whereas that in the inner area was consistent with the hyper-enhancement typical of metastatic lesions. Furthermore, the results of CEUS helped to generate the biopsy strategy.

CEUS is useful in the evaluation of liver metastases. In a meta-analysis that included 828 lesions, the overall sensitivity of CEUS was 91% (4). Several studies have clearly shown that CEUS allows better imaging of the liver, especially in cases with superficial metastases and where there are lesions along the ligamentum teres (5, 6). Similarly, intraoperative CEUS has been shown to have better diagnostic performance than conventional ultrasound for detection of small liver metastases (7). CEUS is also comparable with contrast-enhanced CT and contrast-enhanced MRI in detection of liver metastases (8, 9). The enhancement pattern of liver metastases in the arterial phase is related to the degree of arterial perfusion. Hypervascular metastases, including NENs, have been reported to show a complete hyper-enhancement pattern in the arterial phase and hypo-enhancement and even non-enhancement in the portal and delayed phases (10). This enhancement pattern was also seen in our case.

Most of the relevant literature on use of CEUS for detection of liver metastases has involved patients with a gastroenteric tumor as the primary. Few studies have included patients with pancreatic NEN, for which the imaging features have been unclear (5). We searched PubMed, Embase, and Google Scholar databases to determine whether peritumoral hepatic steatosis was a unique feature of metastatic pancreatic NEN. The search identified nine cases, the clinical and imaging characteristics of which are shown in **Table 1** (11–18). The patients’ ages at the time of diagnosis of metastatic liver lesions were 27–77 years, with a slight female predominance. The time interval between diagnosis of the primary tumor and liver metastases was up to 10 years but mostly less than 5 years. Ultrasonography, CT, and MRI were used to detect metastases in these reports, and our case is the first in which CEUS was used. In terms of imaging features, the fatty infiltration pattern was likely a focal rim surrounding the metastatic tumor in most of the reported cases, including in our case, although there was one report of a wedge-shaped lesion with steatosis distal to the portal flow (11). Most studies did not report the size of the lesions; those that did reported the size of the inner area to be less than 2 cm. In terms of pathological subtype, seven of the nine reported cases were insulinoma and two were non-functioning NEN. Insulin immunostaining was positive for one of the non-functioning NEN and negative for all common hormones, including insulin, gastrin, somatostatin, and glucagon, in the other.

**Table 1:** Brief review of metastatic NEN cases with peritumoral liver steatosis in the literature and this case.

Characteristics	Hoshiba (11)	Sohn (12)	Fregeville (13)	Sumiyoshi (14)	Takeshita (15)	Atwell (16)	Atwell (16)	Borghei (17)	Muramae (18)	This case
Age*	64	27	30	44	69	74	58	54	77	49
**Gender**	F	M	F	F	F	M	M	F	F	F
Time interval**	9 years	NA	2 years	NA	0	0	3 years	0	3 years	4 years
**Pathology**	N-F	Insul	Insul	Insul	Insul	Insul	Insul	N-F	Insul	N-F
Immunohistochemistry***	Insulin	NA	NA	NA	Insulin	NA	NA	All negative	NA	Somat-ostatin
**Imaging procedures**	US/CT	MRI	CT/MRI	US/MRI	CT/MRI	US/CT	US/CT	CT	US/CT/ MRI	MRI/ CEUS
**Imaging feature**	Wedge	Rim	Rim	Rim	Rim	Rim	Rim	Rim	Rim	Rim
**Inner area size**	1cm	1.2cm	NA	NA	0.5cm	NA	NA	2cm	NA	2.2cm
**Overall size**	NA	1.9cm	NA	NA	3cm	NA	NA	NA	NA	6.7cm

M, male; F, female; N-F, non-functioning neuroendocrine neoplasm; Insul, insulinoma; NA, not acquired; US, ultrasound; CT, computed tomography; MRI, magnetic resonance imaging.

*Age at diagnosing liver metastasis.

**Time between the diagnosis of primary tumor and metastasis.

***Common hormones for NEN immunohistochemistry clinically, including insulin, gastrin, somatostatin, and glucagon. NA means the results were not mentioned in the original article, rather than negative.

The mechanism *via* which peritumoral hepatic steatosis develops in patients with metastatic NEN is still uncertain. However, the insulin produced by insulinomas may explain the steatosis around the metastases. Insulin is known to be important in the pathogenesis of hepatic steatosis. A higher level of insulin could inhibit oxidation of free fatty acids and activate genes associated with *de novo* lipogenesis, leading to accumulation of fat in hepatocytes (19). The phenomenon of local steatosis caused by insulin has also been observed in patients with intraperitoneal insulin delivery (20) and those with intraportal islet transplantation (21). In contrast, glucagon facilitates oxidation of fatty acids in hepatocytes and accordingly favors lipolysis (22). There has been a report of a patient with a history of diffuse non-alcoholic fatty liver disease who was found to have metastatic glucagonoma surrounded by a steatosis-deficient zone with an imaging pattern that was also opposite to that of insulinoma (13).

The theory of local hormone production could explain some cases of insulinoma and the reported case of non-functioning NEN with positive insulin staining, which also produced insulin in the area surrounding the tumor (11). However, this theory could not explain our case or the case that produced no hormones. Somatostatin can regulate blood glucose levels under physiological conditions by inhibiting insulin and glucagon secretion from the pancreas (23). However, our patient had undergone total pancreatectomy before liver metastasis was recognized. Furthermore, one report mentioned that a localized decrease in portal flow was one of the causes of focal fatty infiltration (11). Because the imaging features of the lesion in that case were not consistent with those in most of the other reports, this suspicion may also be imperfect. Although we could not confirm the cause of focal steatosis in our patient pathologically, it was speculated that a change in the tumor microenvironment may have contributed to the fatty changes associated with the metastatic NEN.

This research has some limitations. First, somatostatin receptor scintigraphy, such as octreoscan or ^68^Ga-DOTATOC positron emission tomography, was not performed before or after surgery. Therefore, there is a small possibility that micrometastases were missed. Second, the mechanism for the development of the peritumoral steatosis remains unclear.

In summary, this is the first report of peritumoral liver steatosis in a patient with metastatic somatostatin-producing oligosymptomatic NEN. Peritumoral liver steatosis may be a specific pathological and imaging feature observed in metastatic NENs. Awareness of this feature is conducive to an early and accurate diagnosis. CEUS and other imaging procedures may help to distinguish tumor size and guide biopsy of metastases.

## Data availability statement

The original contributions presented in the study are included in the article/Supplementary Material. Further inquiries can be directed to the corresponding authors.

## Ethics statement

Written informed consent was obtained from the individual(s) for the publication of any potentially identifiable images or data included in this article.

## Author contributions

YS and YG contributed equally to this study and should be regarded as co-first authors. YS and YG collected the imaging and clinical data, designed the study, and interpreted the results. YS drafted the manuscript. YG performed the US and CEUS scan. YC followed up the patient and collected the clinical data. JX perform the CT and MRI scans. XC performed the pathological analysis and interpreted the results. KL designed the study, perform the CEUS and biopsy. All authors contributed to the article and approved the submitted version.

## Funding

This study was funded by National Natural Science Foundation (82171968 to KL) and the Chinese Academy of Medical Sciences Innovation Fund for Medical Sciences (CIFMS), CAMS Innovation Fund for Medical Sciences (CIFMS) (award number: 2020-I2M-C&T-B-039 to KL) The funder’s role was collecting the clinical and imaging data, and open access publication fees.

## Conflict of interest

The authors declare that the research was conducted in the absence of any commercial or financial relationships that could be construed as a potential conflict of interest.

## Publisher’s note

All claims expressed in this article are solely those of the authors and do not necessarily represent those of their affiliated organizations, or those of the publisher, the editors and the reviewers. Any product that may be evaluated in this article, or claim that may be made by its manufacturer, is not guaranteed or endorsed by the publisher.
